# Determination of the most suitable reference gene for transcription analysis of synovial tissue from osteoarthritis patients

**DOI:** 10.1038/s41598-026-62328-2

**Published:** 2026-07-17

**Authors:** Diana Freitag, Britt Wildemann, Annett Eitner

**Affiliations:** Experimental Trauma Surgery, Department of Trauma, Hand and Reconstructive Surgery, Jena University Hospital, Friedrich Schiller University Jena, Am Klinikum 1, 07747 Jena, Germany

**Keywords:** Osteoarthritis, qPCR, Reference genes, Synovial tissue, Housekeeping gene, Musculoskeletal system, Analysis, Genetics, Human, Medical research, Osteoarthritis, Pathology, Biomarkers, Computational biology and bioinformatics, Diseases, Genetics, Medical research, Rheumatology

## Abstract

Synovial tissue plays a key role in osteoarthritis (OA) pathogenesis. Gene expression analysis is widely used to investigate underlying pathomechanisms; however, accurate normalization of target mRNA expression depends on the use of stable reference genes. This study evaluated the suitability of common reference genes for determining synovial tissue mRNA expression, considering various factors. In the synovial tissue of 20 patients with end-stage OA, the stability of the expression of 10 reference genes (*GAPDH*,* RPLP0*,* YWHAZ*,* TBP*,* PPIA*,* EEF1A1*,* ACTB*, *HRPT1*, *SDHA*, and *RPL13A*) was evaluated using four different analysis methods (NormFinder, geNorm, the ΔCt method, and Pearson correlation analysis). The most stable genes were identified by means of a subsequent ranking analysis. Both the entire sample pool and various subgroups (sex, age, BMI, and synovitis score) were considered. *RPL13A*,* PPIA*, and *EEF1A1* were identified as the most stable reference genes overall, with minor variability across subgroups. In contrast, *GAPDH* proved to be the least suitable reference gene, showing the most variable expression. The stability of the reference gene might be affected by sex, age, and BMI and this should be taken into account.

## Introduction

Osteoarthritis (OA) is a complex, chronic disease involving inflammatory and destructive processes that lead to various pathological changes, including cartilage degeneration, subchondral bone sclerosis, osteophyte formation, bone marrow lesions, and synovial inflammation^1^. While many studies have examined the pathological processes and changes in cartilage, research into synovial tissue from OA patients is less common. However, recent studies have demonstrated the important role of synovial tissue in disease progression and OA-related pain^2,3^. RT-qPCR-based gene expression analysis is a powerful tool for gaining detailed insight into the pathophysiological processes of synovial tissue. In order to ensure precise analysis of mRNA expression, it is essential to carry out accurate data normalization, for example to an unregulated “stable” gene. However, there is currently no information on reliable unregulated reference genes for studying processes in synovial tissue of OA patients. Since age, sex and obesity are risk factors for OA that can also influence OA processes through senescence, inflammatory mediators and hormones^3–5^, it is standard practice to adjust study results for these factors. Nevertheless, when selecting stable reference genes, the effects of age, sex and BMI on their expression are rarely considered, despite studies demonstrating that these parameters can affect the expression of typical reference genes in various tissues and cells^6–8^. Furthermore, local inflammatory processes and systemic inflammation induced by conditions such as diabetes mellitus or obesity are known to affect intracellular biochemical processes in synovial cells^3^, which may also affect the expression of reference genes. The selection of an appropriate reference gene can significantly impact the results of expression analysis. The utilization of an unregulated reference gene as an internal control for data normalization is therefore essential^9^. Using an inappropriate reference gene in RT-qPCR has the potential to result in the erroneous interpretation of results.

A wide range of mathematical algorithms are available for the evaluation of the usability of reference genes. These include NormFinder, geNorm, Pearson correlation analysis and the ΔCt method. The Microsoft Excel-based software application NormFinder estimates both intra- and intergroup variation in gene expression for each potential gene, combining this to calculate a stability score (S). This is consistent with the measure of systematic error that occurs when using the individual gene for normalisation^10^. GeNorm, an additional Excel-based application, employs the M value to analyse the stability of each reference gene. This is achieved by measuring the average pairwise variation with the other genes. By iteratively excluding the most unstable genes, the two most stable reference genes can be determined^11^. The ΔCt method calculates the ΔCt values of all reference genes in relation to all target genes. This allows for the calculation of standard deviations, which can be used as a measure of stability^12^. Pearson correlation analysis is a method of comparing the gene expression of a specific gene with the average expression of all other reference genes. The inverse correlation coefficient is a measure of stability. The most stable gene can be evaluated by ranking the reference genes of each algorithm using the method implemented by Cheng et al.^13^.

The objective of this study was to validate specific reference genes for the analysis of synovial tissue from OA patients, with a particular focus on examining their stability in specific subgroups related to age, sex, and BMI. Furthermore, the stability of these reference genes in relation to the inflammatory level of the synovial tissue was evaluated. Finally, the most stable reference genes were validated by analysing their impact on target gene expression in comparison to the least stable reference genes.

## Results

### Expression levels of reference genes

The potential reference genes glyceraldehyde-3-phosphate dehydrogenase (*GAPDH*), beta-actin (*ACTB*), ribosomal protein L13a (*RPL13A*), elongation factor1-alpha 1 (*EEF1A1*), tyrosine-3-monooxygenase/tryptophan-5-monooxygenase activation protein (*YWHAZ*), acidic ribosomal protein P0 (*RPLP0*), peptidylprolyl isomerase A (*PPIA*), TATA-binding protein (*TBP*), hypoxanthine phosphoribosyl transferase 1 (*HPRT1*) and succinate dehydrogenase complex, subunit A (*SDHA*) were tested to determine their suitability for normalisation of target genes in human synovial tissue.

**Fig. 1:**
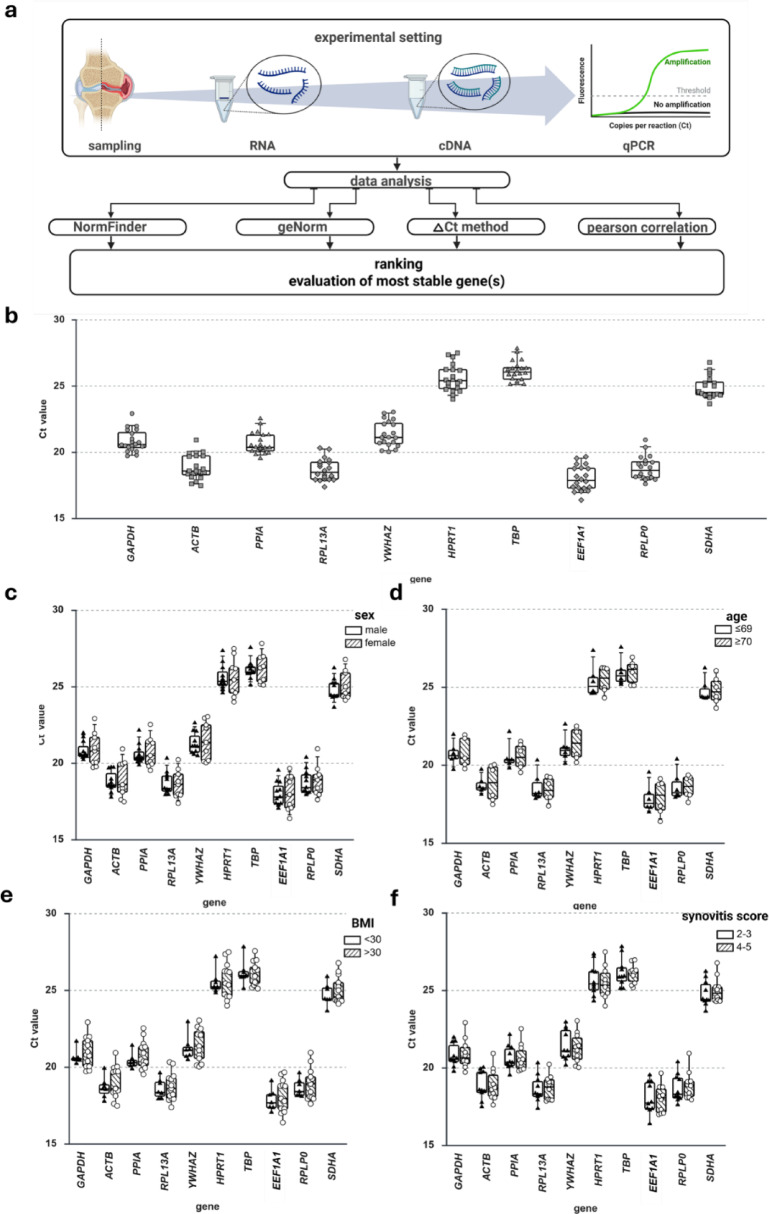
Experimental design (created in BioRender. Freitag, D. (2026). https://BioRender.com/q0s68vm*)* (**a**) and expression levels (Ct values) of all pre-selected genes for internal normalization (**b**) –(**e**). The graphs show gene expression with the variation of all Ct values for total sample pool (**b**), subgrouped by sex (**c**), age (**d**), BMI (**e**) and synovitis score (**f**). The boxes show the IQR 25–75% (box), the median value (line) and the CI of 5–95% (error bars) (**a-e**) were created in https://BioRender.com.

Analysis of the Ct values revealed large differences in the expression levels of the candidate genes. The mean Ct values for the various reference genes ranged from 18.07 (*EEF1A1*) to 26.15 (*TBP*). Figure 1b shows the respective Ct values for all ten candidate reference genes tested in all synovial samples, with a direct comparison of their transcription levels. The most highly expressed genes were: *EEF1A1* (Ct = 18.07 ± 0.91), *RPL13A* (Ct = 18.70 ± 0.80), *RPLP0* (Ct = 18.83 ± 0.87) and *ACTB* (Ct = 18.91 ± 0.92). The lowest expressions were detected for *TBP* (Ct = 26.15 ± 0.74) and *HPRT1* (Ct = 25.63 ± 0.99). The remaining genes

examined showed moderate expression, with Ct values ranging between 20 and 25. Subgrouping by age, sex, BMI, and synovitis score revealed no significant differences in Ct values between subgroups (Fig. 1c-f).

### Expression stability and validation of reference genes of the total sample set

**Fig. 2:**
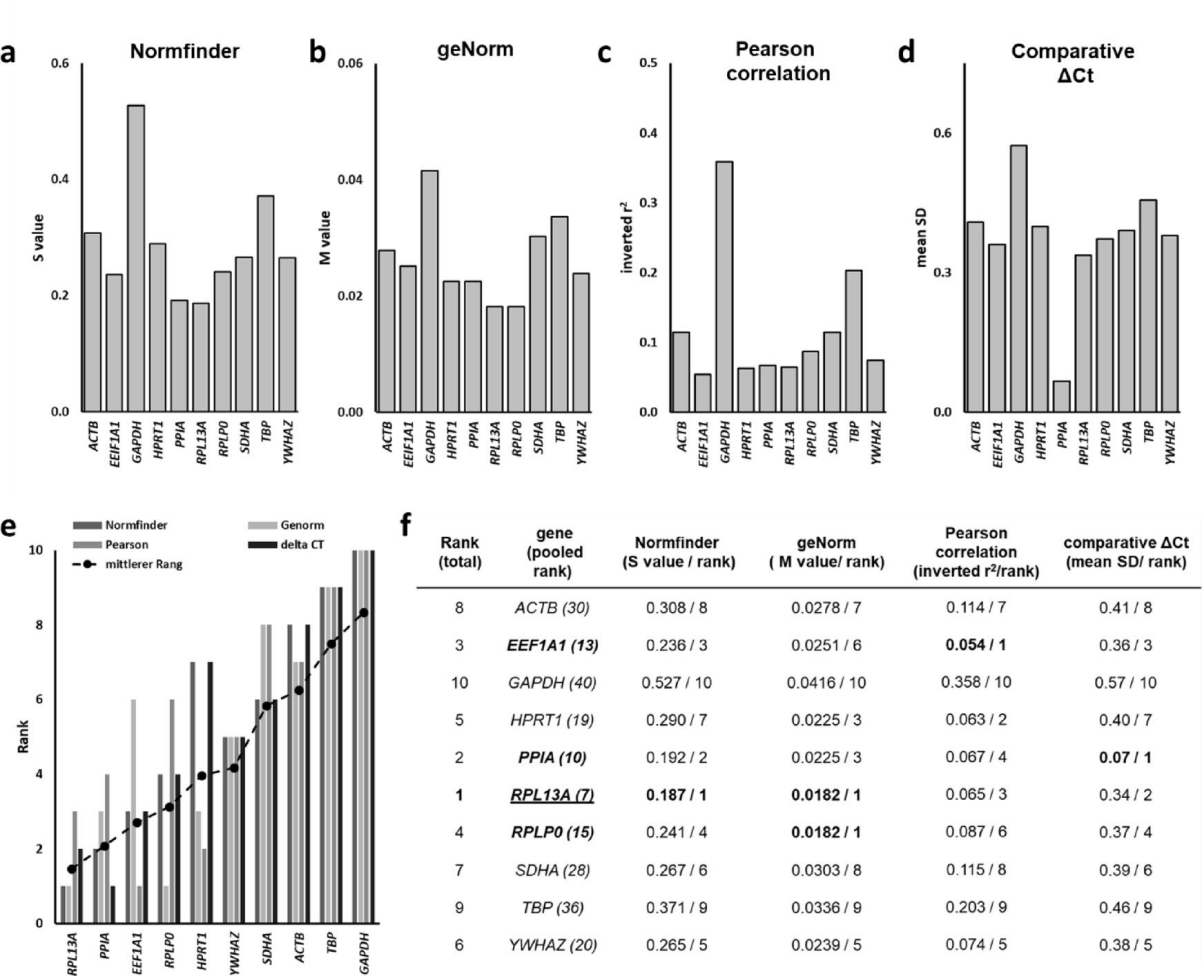
Reference gene analysis of total sample set. The graphs display (**a**) the results of the NormFinder analysis with the stability values [S], (**b**) the results of the geNorm analysis with the M value, (**c**) the results of the Pearson correlation by the inverted coefficient of correlation (r^2^) and (**d**) the results of the ΔCt method indicating the mean standard deviation. (**e**) demonstrates the results of the ranking analysis as well as (**f**) the detailed listed results of each analysis algorithm.

The analysis of stability involved the usage of geNorm, NormFinder, the ΔCt method and Pearson’s correlation, with the calculation of an overall ranking. Each of these algorithms is designed to evaluate gene stability from a specific perspective. The NormFinder software employs a statistical approach to evaluate stability, accounting for variances both within and between groups. The most stable genes were *RPL13A* (S = 0.187) and *PPIA* (S = 0.192), whereas *TBP* (S = 0.371) and *GAPDH* (S = 0.527) demonstrated the least stability (Fig. 2a, e).

The geNorm algorithm is an Excel-based tool that identifies stable reference genes by progressively excluding unstable genes and calculates the stability value M thereby identifying the most stable reference gene pair with the lowest M value^11^. *RPLP0* and *RPL13A* (both M = 0.182) were identified as the most stable candidates (Fig. 2b, e).

Furthermore, a Pearson correlation was performed based on unnormalised values, whereby a low inverted correlation coefficient is associated with higher stability. For the total sample pool, the coefficient of determination for *EEF1A1* was 0.946 (*p* < 0.001), indicating that 94.6% of the data representing *EEF1A1* expression corresponded to the expression of the remaining nine reference genes within the group studied. The inverted coefficient of determination for all reference genes are displayed in Fig. 2c, values approaching 0 define stable genes, whereas values exceeding 0.5 indicate less stable genes (Fig. 2c, e). Consequently, *EEF1A1* exhibited the highest degree of conformity, indicating its optimal stability.

The final method for evaluating the most suitable reference gene was the ΔCt method^12^. The method involved the comparative analysis of the relative expression of gene pairs within each sample. A comparison of the standard deviation of the calculated ΔCt values between the different samples was used to determine whether a gene was stably expressed or co-regulated. This approach took into account all reference genes and compared all possible gene combinations within each sample group. Following the estimation of the mean standard deviation (mean SD), it was determined that *PPIA* (mean SD_total_ = 0.07) exhibited the lowest level of variability, while *GAPDH* (mean SD_total_ = 0.57) demonstrated the highest level of variability (Fig. 2d, e).

In conclusion, the ranking analysis determined that *RPL13A*,* PPIA* and *EEF1A1* were the most stable genes across four different analyses. *RPL13A* demonstrated rankings ranging from 1 to 3, thus being identified as the most stable. *GAPDH*,* TBP* and *ACTB* were identified as the least stable. Notably, both *GAPDH* (rank 10) and TBP (rank 9) were consistently assigned the least stable genes in all analyses (Fig. 2f).

### Expression stability and validation of reference genes after subgrouping

In order to assess the influence of co-factors, the dataset was stratified by sex, age, BMI and synovitis score, and subsequently reanalysed (Table 1).

**Fig. 3:**
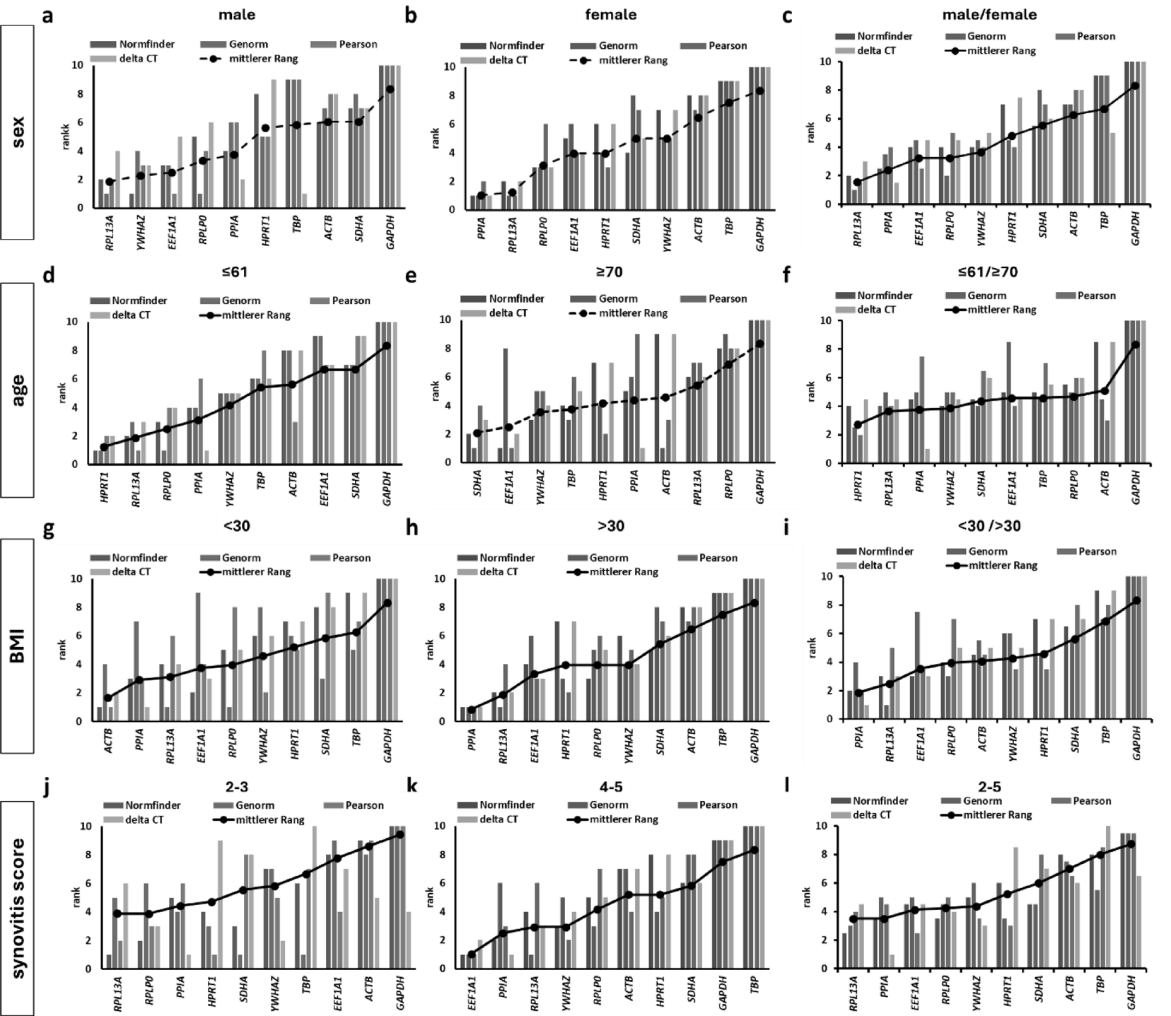
Ranking of subgroup analysis. The graphs show the result of the final ranking analysis of all four processed evaluation methods for all subgroups: (**a**) male, (**b**) female, (**c**) mixed sex group, (**d**) age ≤ 61, (**e**) age ≥ 70 (f) mixed age group, (**g**) BMI < 30, (**h**) BMI > 30, (**i**) mixed BMI group, (**j**) synovitis score 2–3, (**k**) synovitis score 4–5 and mixed synovitis score group. All mixed groups (sex, age, BMI, synovitis score) are taking into account their individual group division.

Firstly, the stability of expression and validation of reference genes were performed following subdivision according to biological sex. *RPL13A* was found to be stable when no sex differentiation was made (Fig. 3c). However, in the subgroup analysis *RPL13A* was the most stable gene in males and *PPIA* in females (Fig. 3a, b). *GAPDH* was the least stable gene overall and in the subgroups (Table 1; Fig. 3a-c).

The second subgrouping was age-based, with an eight-year gap (62–69 years), to clearly distinguish between younger and older patients. The analysis identified *HPRT1* as most stable across both groups and in the ≤ 61 years subgroup. Conversely, *SDHA* was identified as most suitable in subjects aged ≥ 70. *GAPDH* was consistently identified as the least stable gene (Table 1; Fig. 3d-f).

A further subdivision was performed based on BMI values. Subsequent analysis identified *PPIA* as most stable across both subgroups and in the BMI > 30 subgroup. For samples from patients with a lower BMI, *ACTB* was found to be the most stable gene. As previously observed, *GADPH* was identified as the least stable under all conditions (Table 1; Fig. 3g-i).

**Table 1:** Results of the subgroup analysis. Listed are the respective method-specific stability values with the corresponding ranks determined for the individual subgroups as well as for the analysis of all analysis methods and consideration of the subgroups (overall). The best gene stability value in each method is underlined. Additionally, the gene with the best rank sum in the first subgroup is underlined and marked with an asterisk (*),  and the gene with the best total rank sum in the second subgroup is marked with a hash (#).

reference-gene	NormFinder		geNorm		pearson correlation		comparative ΔCt		total	NormFinder		geNorm		pearson correlation		comparative ΔCt		total	overall final rank
	S value	rank	M value	rank	inverted *r*^2^	rank	mean SD	rank	rank sum	S value	rank	M value	rank	inverted *r*^2^	rank	mean SD	rank	rank sum	
sex (*n* = 20)																			
*male (n = 10)*										*female (n = 10)*									
RPL13A*	0.152	2	0.013	1	0.049	2	0.271	4	9*	0.171	2	0.017	1	0.034	1	0.367	2	6	1
*PPIA#*	0.224	4	0.023	6	0.119	6	0.034	2	18	0.150	1	0.017	1	0.036	2	0.133	1	*5#*	2
*EEF1A1*	0.168	3	0.017	3	0.035	1	0.274	5	12	0.307	5	0.030	6	0.061	4	0.427	4	19	3
*RPLP0*	0.230	5	0.013	1	0.074	4	0.307	6	16	0.269	3	0.020	3	0.080	6	0.424	3	15	3
*YWHAZ*	0.146	1	0.021	4	0.054	3	0.269	3	11	0.367	7	0.028	5	0.074	5	0.463	7	24	5
*HPRT1*	0.262	8	0.021	5	0.083	5	0.331	9	27	0.320	6	0.022	4	0.042	3	0.447	6	19	6
*SDHA*	0.249	7	0.024	8	0.133	7	0.318	7	29	0.275	4	0.036	8	0.082	7	0.432	5	24	7
*ACTB*	0.242	6	0.024	7	0.136	8	0.323	8	29	0.377	8	0.032	7	0.087	8	0.473	8	31	8
*TBP*	0.288	9	0.028	9	0.188	9	0.000	1	28	0.472	9	0.041	9	0.211	9	0.549	9	36	9
*GAPDH*	0,358	10	0.030	10	0.270	10	0.399	10	40	0.707	10	0.054	10	0,387	10	0.720	10	40	10
age (*n* = 12)																			
*Age* ≤ *61 (n = 6)*										*age* ≥ *70 (n = 6)*									
HPRT1*	0.071	1	0.010	1	0.029	2	0.325	2	6*	0.291	7	0.019	4	0.030	2	0.337	7	20	1
*RPL13A*	0.087	2	0.015	3	0.018	1	0.334	3	9	0.246	6	0.027	7	0.066	7	0.301	6	26	2
*PPIA*	0.195	4	0.018	4	0.075	6	0.167	1	15	0.244	5	0.022	6	0.097	9	0.057	1	21	3
*YWHAZ*	0.310	5	0.021	5	0.075	5	0.392	5	20	0.156	3	0.020	5	0.044	5	0.285	4	17	4
*SDHA#*	0.378	7	0.040	7	0.198	9	0.478	9	32	0.154	2	0.011	1	0.043	4	0.278	3	*10#*	5
*EEF1A1*	0.424	9	0.041	9	0.085	7	0.458	7	32	0.147	1	0.028	8	0.015	1	0.268	2	12	6
*TBP*	0.315	6	0.031	6	0.156	8	0.437	6	26	0.197	4	0.017	3	0.061	6	0.290	5	18	6
*RPLP0*	0.183	3	0.010	1	0.054	4	0.362	4	12	0.292	8	0.030	9	0.096	8	0.346	8	33	8
*ACTB*	0.414	8	0.041	8	0.053	3	0.462	8	27	0.308	9	0.011	1	0.040	3	0.363	9	22	9
*GAPDH*	0.836	10	0.062	10	0.703	10	0.795	10	40	0.466	10	0.036	10	0.307	10	0.465	10	40	10
BMI (*n* = 20)																			
*BMI < 30 (n = 7)*										*BMI > 30 (n = 13)*									
*PPIA#*	0.199	3	0.023	7	0.068	3	0.048	1	14	0.154	1	0.019	1	0.041	1	0.094	1	*4#*	1
*RPL13A*	0.201	4	0.010	1	0.116	6	0.296	4	15	0.176	2	0.019	1	0.049	4	0.339	2	9	2
*EEF1A1*	0.191	2	0.023	9	0.079	4	0.290	3	18	0.265	4	0.028	6	0.049	3	0.381	3	16	3
*RPLP0*	0.214	5	0.010	1	0.126	8	0.300	5	19	0.264	3	0.025	5	0.075	6	0.394	5	19	4
ACTB*	0.139	1	0.019	4	0.057	1	0.276	2	8*	0.366	8	0.031	7	0.126	8	0.450	8	31	5
*YWHAZ*	0.263	6	0.023	8	0.065	2	0.336	6	22	0.278	6	0.023	4	0.072	5	0.391	4	19	6
*HPRT1*	0.270	7	0.022	6	0.102	5	0.342	7	25	0.300	7	0.019	3	0.044	2	0.409	7	19	7
*SDHA*	0.288	8	0.017	3	0.171	9	0.343	8	28	0.265	5	0.034	8	0.092	7	0.400	6	26	8
*TBP*	0.308	9	0.021	5	0.117	7	0.364	9	30	0.367	9	0.037	9	0.139	9	0.455	9	36	9
*GAPDH*	0.425	10	0.033	10	0.408	10	0.444	10	40	0.588	10	0.046	10	0.343	10	0.620	10	40	10
synovitis score (n = 20)																			
*synovitis score 2–3 (n = 10)*										*synovitis score 4–5 (n = 10)*									
RPL13A*	0.136	1	0.023	5	0.041	2	1.058	6	14*	0.229	4	0.015	1	0.079	6	0.323	3	14	1
*PPIA*	0.241	5	0.022	4	0.088	6	0.413	1	16	0.145	2	0.022	6	0.041	3	0.064	1	12	1
*EEF1A1#*	0.309	8	0.033	9	0.062	4	1.175	7	28	0.141	1	0.015	1	0.032	1	0.295	2	*5#*	3
*RPLP0*	0.188	2	0.024	6	0.058	3	1.025	3	14	0.245	5	0.020	3	0.083	7	0.347	5	20	4
*YWHAZ*	0.287	7	0.026	7	0.082	5	0.934	2	21	0.197	3	0.021	5	0.037	2	0.325	4	14	5
*HPRT1*	0.237	4	0.020	3	0.036	1	1.479	9	17	0.300	8	0.020	4	0.059	5	0.377	8	25	6
*SDHA*	0.237	3	0.015	1	0.092	8	1.303	8	20	0.249	6	0.029	8	0.095	8	0.354	6	28	7
*ACTB*	0.365	9	0.032	8	0.170	9	1.049	5	31	0.261	7	0.026	7	0.056	4	0.355	7	25	8
*TBP*	0.257	6	*0.015*	*1*	0.090	7	1.587	10	24	0.469	10	0.040	10	0.248	10	0.498	10	40	9
*GAPDH*	0.637	10	0.048	10	0.504	10	1.045	4	34	0.421	9	0.033	9	0.198	9	0.464	9	36	10

BMI: body mass index; SD: standard deviation.

The final subgrouping was based on the histologically determined synovitis score. *RPL13A* was identified as the most stable reference gene across the subgroups and in samples with a low (2–3) synovitis score. *EEF1A1* was the most stable gene for samples with higher scores. *TBP* was identified as the least stable reference gene in the group with a higher synovitis score, whereas *GAPDH* was identified as the least stable in the other subgroup and across both groups. (Table 1; Fig. 3j-l)

In all subgroup analyses, different results were found between the respective subgroups when different evaluation methods were used to assess the stability of the reference genes.

In summary, *RPL13A* was one of the most stable reference genes in the entire sample set and in almost all subgroups. *GAPDH* was always the least stable gene in all subgroups, with one exception (Table 1).

### Expression of target genes

**Fig. 4:**
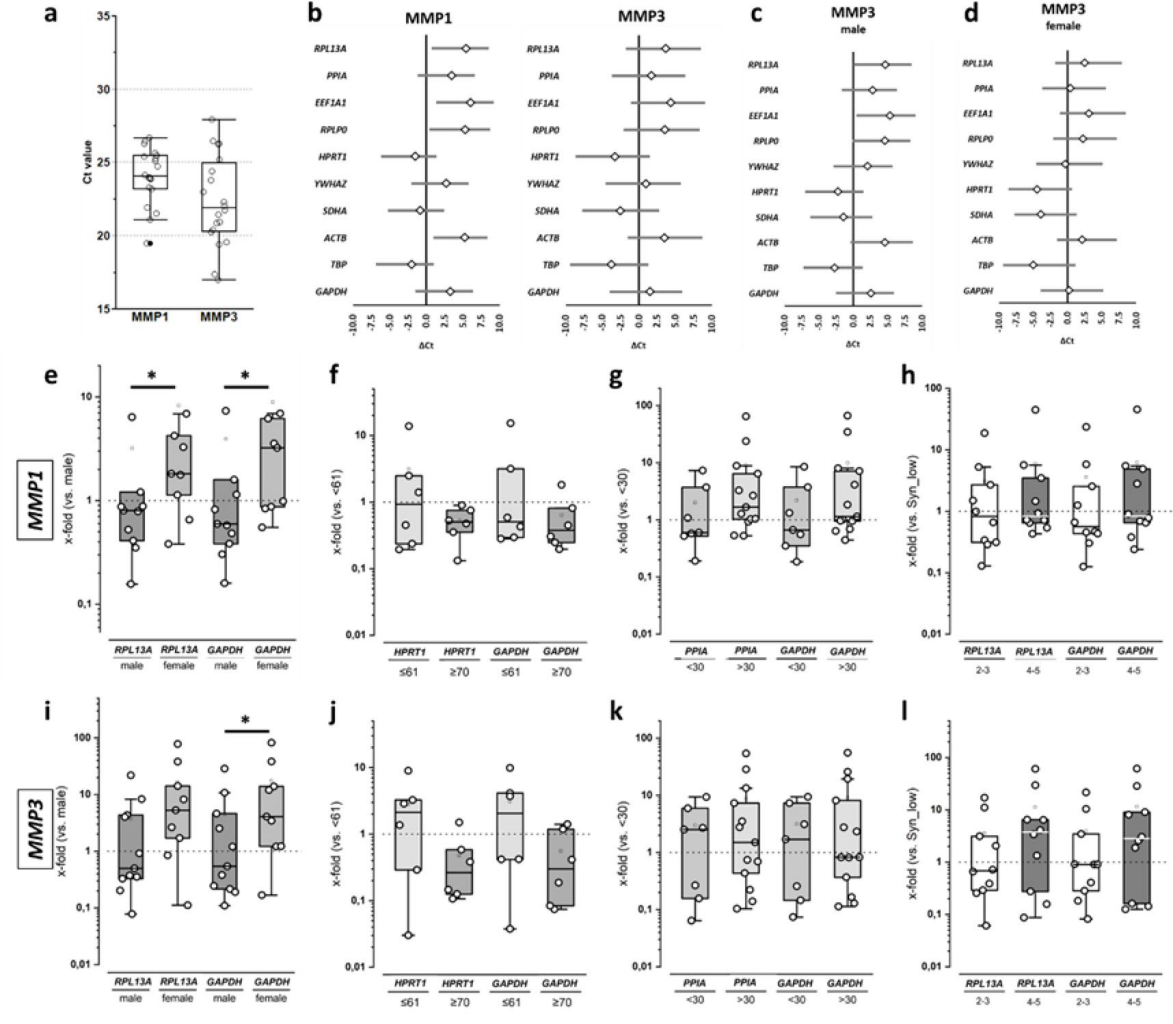
Target gene analysis of *MMP1* and *MMP3*. The graphs display (**a**) Ct values of *MMP1* and *MMP3*, (**b**) differences between target and reference genes for *MMP1* and *MMP3* and the sex-specific subgroups male (**c**) and female (**d**) for *MMP3* as well as the results of the expression analysis calculated by ΔΔCt method for the genes *MMP1* (**e-h**) and *MMP3* (**i-l**), normalised to the most stable and weakest genes, respectively, in comparison after subgrouping by sex (**e**,** i**), age (**f**,** j**), BMI (**g**,** k**), and synovitis score (**h**,** l**). Mann-Whitney U test, *p ≤ 0.05.

*MMP1* and *MMP3* are produced by synovial cells in osteoarthritis and contribute to cartilage degradation and elevated levels correlate with disease onset and severity^14–16^. The averaged Ct values were 24.07 (± 1.86) for *MMP1* and 22.33 (± 2.98) for *MMP3* (Fig. 4a). The differences in the normalization of *MMP1* and *MMP3* to all reference genes is shown in Fig. 4b. The effect of sex on this normalization is exemplary shown for MMP3 and all reference genes. The greatest similarity was found between *MMP3* normalized to *YWHAZ*,* PPIA*, and *GAPDH* (Fig. 4c).

For normalisation of *MMP1* and *MMP3* expressions in the specific subgroups *HPRT1* (subgroup age), *PPIA* (BMI) and *RPL13A* (sex and synovitis score) were used as stable reference genes, whilst *GAPDH* was used as less stable (Fig. 4).

Differential expression of *MMP1* was observed according to sex-specific separation, independent of the reference gene employed for normalisation. A significantly higher *MMP1* expression was observed in female samples independent of the reference gene (1.8-fold normalised with GAPDH, *p* = 0.044; 1.7-fold with RPL13A, *p* = 0.037) (Fig. 4e). No significant differences were observed in the subgroups age, BMI, or synovitis score. (Fig. 4f-h).

For *MMP3*, inconsistent results were obtained in the sex-specific separation depending on the reference gene: a significant difference was identified with *GAPDH* (*p* = 0.044), whereas using *RPL13A*, no significant difference was observed (Fig. 4i). With regard to the subgroups based on BMI, age, and synovitis score, no significant differences between the respective subgroups could be determined (Fig. 4j-l).

## Discussion

The expression level of genes can vary significantly in human native tissue depending on disease, disease severity, comorbidities, and parameters such as age, sex and BMI. In context of analysing native human tissue, selection of an appropriate reference gene is crucial, especially in context of group comparisons or in cases of highly heterogeneous study groups^17^. Reference genes are utilised to normalise target gene expression, thereby minimising technical variability and reducing the risk of misinterpretation^18,19^. Consequently, the selection of inappropriate reference genes can result in misinterpretation of data^9,20^. Ideally, reference genes should exhibit stable expression across all experimental conditions^19,21^.

Alterations in gene expression have been demonstrated in synovial tissue/cells of OA or RA patients^22^. However, different reference genes for normalisation had been used, such as genes that influence metabolism, structural proteins or ribosomal activity^23–28^. These include genes that code for glyceraldehyde-3-phosphate dehydrogenase (*GAPDH*), beta action (*ACTB*) and ribosomal RNA (rRNA)^18,23–25,29,30^.

A limited number of studies have systematically evaluated suitable reference genes in synovial tissue or cells. These identified alternatives include *HPRT1*,* RPLP0*, and *EEF1A1*^27,28^. It is important to note that Watanabe et al. remains the only study in which such an analysis was performed with human synovial tissue from knee OA patients^28^, but they did not considered effects of demographic parameters on the reference gene stability.

The present study aimed to identify suitable reference genes for human synovial tissue from OA patients, considering age, BMI, sex, and inflammation. The selection of ten candidate genes was made on the basis of their functional diversity and frequent use in gene expression studies: *GAPDH*^29,31^, *RPLP0*^27^, *YWHAZ*^32^, *TBP*^32,33^, *PPIA*^32^, *EEF1A1*^27^, *ACTB*^24,25^, *HRPT1*^28,30^, *SDHA*^26^, and *RPL13A*^33^. These genes are extensively utilised in both in vitro and ex vivo contexts. Our analysis identified *RPL13A*,* PPIA*, and *EEF1A1* as the most stable reference genes across the entire cohort, although rankings varied slightly depending on the algorithm applied. Conversely, *GAPDH* and *TBP* exhibited the least stability.

These findings are consistent with those of previous studies. The eukaryotic translation elongation factor 1 alpha 1 *EEF1A1* gene has been described as highly stable in synovial fibroblasts of OA and non-OA patients^27^ and is widely recommended due to its ubiquitous expression^34,35^. In accordance with the results of our study, *RPL13A*, which encodes the 60S ribosomal protein L13a, has been identified as a reliable reference gene for human OA articular cartilage^36^, for hypoxia-cultured human chondrocytes^37^, and for arthritic tissue from experimental models^33^. The third suitable reference gene that was identified in the present study is *PPIA*, which encodes peptidylprolyl isomerase A. *PPIA* has also been recommended in synovial and mesenchymal stem cells^38^. Conversely, *GAPDH* has been repeatedly reported as unstable despite its extensive utilisation^23,29,31,36,37,39^.

In general, the evaluated genes exhibited high stability, with the majority of the stability values falling below 0.5, indicating a high overall level of stability. In comparison with cartilage, synovial tissue appears to exhibit higher reference gene stability^27,28^. Studies analysing cartilage or cultured chondrocytes have shown substantially higher stability values, indicating lower stability^36,37^. Nevertheless, the suitability of a reference gene remains dependent on tissue type, disease characteristics, and cohort composition^6^.

The observed variations between studies can be attributed to the distinct characteristics of the cohorts involved. For instance, Watanabe et al. identified *HPRT1* as the most stable gene, whereas in our cohort it ranked lower^28^. However they analysed tissue from patient of older age and lower BMI compared to our study, which might explain the differences. As demonstrated earlier, *HPRT1* exhibited elevated levels of variability in aged subjects^7,8^.

Subgroup analyses in our study further demonstrated that reference gene stability depends on patient characteristics. *HPRT1*,* RPL13A*, and *PPIA* exhibited the greatest stability in younger patients, whereas *SDHA* and *EEF1A1* demonstrated superior performance in older individuals. These findings provide further evidence in support of previous reports of age-dependent variability, particularly with regard to *HPRT1* and *ACTB*^8^.

Furthermore, sex-specific differences have been demonstrated to influence gene stability. While *RPL13A*,* PPIA*, and *EEF1A1* demonstrated consistent stability overall, subgroup analysis revealed fluctuations. *RPL13A*,* YWHAZ*, and *EEF1A1* exhibited the greatest stability in males, while *PPIA*,* RPL13A*, and *RPLP0* demonstrated the highest rankings in females. This finding is consistent with the documented sex-related differences in gene expression and treatment responses, as previously outlined by Mauvais-Jarvis et al.^40^.

Subgroup analyses regarding BMI and inflammation levels identified *RPL13A*,* PPIA*, and *EEF1A1* as the most stable genes, with slight changes observed in the separate analysis of single subgroups.

Similarly, BMI and inflammation influenced stability rankings, although core genes (*RPL13A*,* PPIA*,* EEF1A1*) remained robust. Notably, *ACTB* performed poorly overall but ranked highest in patients with BMI < 30, illustrating the importance of cohort composition. Many studies do not explicitly address such variability or clearly justify reference gene selection^25,31,41^.

In addition to the utilisation of algorithm-based stability assessment methodologies, it is imperative to consider the impact of other pertinent factors. These include the stable efficiency of the reference genes^42,43^, the use of 2–3 different reference genes (geometric mean), which significantly reduces error^11,44^, and the expression strength of the target gene. Its proximity to the reference gene is also important to avoid extreme extrapolation of the values^45^. However, the use of a single reference gene is permissible if the variations have been previously investigated and the differences between the reference genes tested are not statistically significant^19^. In this study, the use of a single reference gene is justified, as no significant differences between candidates were observed.

A detailed analysis of the OA mediators *MMP1* and *MMP3* revealed that the majority of reference genes exhibit sufficient stability to serve as reliable reference. The Ct values of *MMP1* ranged from 20 to 27, while *MMP3* showed a wider range from 17 to 28. Consequently, all reference genes examined appear to be suitable for the analysis of *MMP1*. However, the standardisation of *MMP3* expression was more challenging due to the wider range. Female samples exhibited a greater proximity to the reference gene than male samples. It is hypothesised that these discrepancies may be responsible for the observed differences in the statistical results. The high stability of *RPL13A* in both male and female samples suggests that this gene is the most suitable reference gene.

The ΔΔCt analysis demonstrated that the majority of the reference genes yielded comparable results across the age, BMI, and inflammation subgroups. However, sex-specific differences were evident: The *GAPDH*-based normalisation suggested significant differences in *MMP3* expression, whereas *RPL13A* did not. This emphasises the potential for misinterpretation when employing unstable reference genes.

The MIQE guidelines (Minimum Information for Publication of Quantitative Real-Time PCR Experiments)^46^ were largely adhered to, ensuring the reproducibility and transparency of the qPCR results. Most of the essential MIQE requirements were met, including detailed descriptions of sample preparation, RNA quality, reverse transcription, primer design, amplification efficiencies, melting curve analyses, reference gene stability and data analysis. However, it was not possible to check for genomic DNA contamination due to methodological limitations. The provided information allows for transparent evaluation and reproduction of experiments.

The study is subject to several limitations. The study’s exclusive focus on OA synovial tissue may limit the generalisability of its findings to non-OA conditions^27^. Furthermore, the preselection of candidate genes may have resulted in the exclusion of superior alternatives. The number of donor tissue in each subgroup was limited, and the definitions employed may have influenced the outcomes. Furthermore, the primer pair utilised for *MMP1* demonstrated an efficiency of 120% in the analysed tissue, which may suggest suboptimal assay performance. Furthermore, RNA integrity numbers (RIN) were marginally below the recommended threshold in some samples, which may be indicative of minor compromises in RNA quality. Nevertheless, it is considered unlikely that the elevated primer efficiency and the slightly reduced RIN values would have had a direct effect on the overall analyses or the interpretation of the results. Nevertheless, the employment of a multi-methodological analytical approach serves to enhance the robustness of the findings^42^.

In conclusion, *RPL13A*,* PPIA*, and *EEF1A1* were identified as the most stable reference genes for OA patients’ synovial tissue, while *GAPDH* was identified as the least suitable. The selection of reference genes has been shown to exert a significant influence on the results of gene expression analyses, particularly in the context of subgroup analyses. Consequently, cohort characteristics such as age, sex, and BMI should be carefully considered in experimental design.

## Methods

### Human materials and samples

Synovial tissue samples were obtained from 20 patients (9 females/ 11 males) with end-stage OA who underwent knee arthroplasty. Patients were on average 66.7 years old (± 7.0 SD; standard deviation) with a mean BMI of 32.2 (± 4.5). For analysis, patient samples were separated in various subgroups (Table 2). The first subgrouping was based on the sex of the patients (male *n* = 11 and female *n* = 9). The second subgrouping was based on age, separating the samples into patients who were aged younger than 61 years (≤ 61 years; *n* = 6) and those who were aged 70 years or older (≥ 70 years; *n* = 6) at the time of sampling. In this specific subgroup analysis, eight patients aged between 61 and 70 years were excluded. The third division was based on body mass index (BMI) into samples from patients with a BMI of less than 30 (< 30; *n* = 7) and a BMI of 30 or above (> 30; *n* = 13). The final subgrouping was based on the histologically determined synovitis score into groups 2–3 (*n* = 10) and 4–5 (*n* = 10) (Table 2). The patients were informed about the purpose of the tissue collection, with all procedures explained to them in detail. Written informed consent was obtained from all subjects and/or their legal guardians. The study was approved by the Ethical Committee for Clinical Trials of the Friedrich-Schiller-University of Jena (*2020-1630-BO*,* 2018 − 1158*) and was conducted in accordance with the Declaration of Helsinki.

**Table 2:** Overview of patient data (age, sex, BMI, and synovitis score) for the total sample set and individual subgroups. The mean values with standard deviation; Fisher’s exact test (exact Chi^2^ test) significant *p* ≤ 0.05.

					distribution [*n*]							
		counts	age	BMI	gender		BMI		age		synovitis score	
group	sub-group	*n*	mean	mean	[♂/♀]	Chi^2^	[< 30/>30]	Chi^2^	[≤ 61/≥70]	Chi^2^	[2–3/4–5]	Chi^2^
**total**		20	66.7 ± 7.0	32.2 ± 4.5	11 / 9	-	7 / 13	-	6 /6	-	10 /10	-
**gender**	***male***	11	66.8 ± 6.8	29.4 ± 2.2	11 / 0	-	6 / 5	*p* = 0.070	3 / 4	*p* = 1.000	7 / 4	*p* = 0.370
	***female***	9	66.4 ± 7.2	35.7 ± 4.2	0 / 9		1 / 8		3 / 2		3 / 6	
**age**	**≤** ***61y***	6	59.2 ± 5.0	34.1 ± 2.3	3 / 3	*p* = 1.000	2 / 4	*p* = 1.000	6 / 0	-	4 / 2	*p* = 1.000
	**≥ 70** ***y***	6	74.8 ± 5.0	31.6 ± 2.3	4 / 2		1 / 5		0 / 6		3 / 3	
**BMI**	***< 30***	7	65.2 ± 4.6	28.0 ± 1.8	6 / 1	*p* = 0.070	7 / 0	-	2 / 1	*p* = 1.000	4 / 3	*p* = 1.000
	***> 30***	13	67.5 ± 7.9	34.5 ± 3.9	5 / 8		0 / 13		4 / 5		6 / 7	
**synovitis score**	***2–3***	10	66.4 ± 7.8	31.1 ± 4.6	7 / 3	*p* = 0.370	4 / 6	*p* = 1.000	4 / 3	*p* = 1.000	10 / 0	-
	***4–5***	10	66.9 ± 6.1	33.4 ± 4.2	4 / 6		3 / 7		2 / 2		0 / 10	

BMI: body mass index; y: year.

After surgical removal, the synovial tissue was immediately frozen in liquid nitrogen and stored at -80 °C for RNA extraction. A second part of the tissue was fixed and prepared for histological evaluation of synovitis as previous described^2^. The inflammation of the synovial tissue was scored according to Krenn^47^. This synovitis score was used in many histological studies to determine the synovial inflammation in OA patients^2,48^. The synovitis score ranges from 0 to 9 (0–1: no synovitis; 2–3: slight synovitis, 4–6 moderate synovitis, 7–9 strong synovitis^47^.

### Total RNA extraction, quantification and reverse transcription

Total RNA was isolated from frozen human tissue samples using the RNAeasy^®^ Mini Kit (Qiagen GmbH, Hilden, Germany) according to the manufacturer’s protocol.

The RNA concentration and quality of isolated RNA were determined spectrophotometrically (NanoDrop 2000, Thermo Fisher Scientific, Wilmington, USA). Samples with a concentration of at least 100 ng/µl RNA were used for synthesis. Complementary DNA synthesis of 100 ng RNA was performed using the qoScript cDNA Synthesis Kit (Quantabio, Beverly, MA, USA) with a volume of 20 µl per batch according to the manufacturer’s instructions.

*Reference and target genes*.

Ten different possible reference genes were tested in synovial tissue with respect to different parameters (age, sex, BMI, synovitis score). These subsets of reference genes tested represent multiple functional categories, reducing the possibility of co-regulation and false-positive gene selection. We tested *glyceraldehyde-3-phosphate dehydrogenase (GAPDH)*,* beta-actin (ACTB)*,* ribosomal protein L13a (RPL13A)*,* elongation factor1-alpha 1 (EEF1A1)*,* tyrosine-3-monooxygenase/tryptophan-5-monooxygenase activation protein (YWHAZ)*,* acidic ribosomal protein P0 (RPLP0)*,* peptidylprolyl isomerase A (PPIA)*,* TATA-binding protein (TBP)*,* hypoxanthine phosphoribosyl transferase 1 (HPRT1)* and *succinate dehydrogenase complex*,* subunit A (SDHA*) (Table 3).

Two target genes, *matrix metalloproteinase 1 and 3 (MMP1*,* MMP3)*, were selected to verify the results of the determined reference gene. These enzymes play an important role in the progression of OA^14,16,49^(Table 3).

### Primer design and quantitative PCR

The human-specific primers for qPCR were designed based on mRNA coding sequences (GenBank) using NetPrimer (Premier BioSoft, Palo Alto, CA, USA; http://www.premierbiosoft.com/netprimer/) and NCBI Primer-BLAST (http://www.ncbi.nlm.nih.gov/tools/primer-blast/; sequences in Table 3) and checked for hairpins or other secondary structures^17,50^. The specificity of amplification was analysed via gel electrophoresis and melting curve analysis. To verify primer specificity the PCR products were analysed using agarose gel electrophoresis to check for the presence of a single size-specific lane. Melting curve analysis also confirmed the purity of the amplificated.

Quantitative PCR (qPCR) was performed in a 10 µl reaction mixture containing PerfeCTa SYBR Green FastMix (Quantabio, Beverly, MA, USA), specific primers at a final concentration of 300 nM each, and cDNA (2.5 ng of cDNA corresponding to reverse transcribed RNA). The specific transcripts of each sample were amplified in triplicate in Rotor-Gene G (Qiagen GmbH) under the following conditions: 5-minute polymerase activation, 40 amplification cycles (95 °C for 10 s, 57 °C for 15 s, 72 °C for 20 s).

**Table 3:** The selected reference and target genes, their amplicon size, primer sequences, accession number, function and efficiency.

Gene (amplicon size)	Full name	Primer sequences (5`- 3`)	Accession number	Function	Efficiency
*reference genes*					
*ACTB* (109 kb)	Beta-actin	F: CTCTTCCAGCCTTCCTTCCT R: TGTTGGCGTACAGGTCTTTG	NM_001101.5	structure protein. formation of cellular cytoskeleton	1.97 (97%)
*EEF1A1* (192 kb)	Eukaryotic translation elongation factor 1-alpha 1	F: TGCCTGGGTCTTGGATAAAC R: ACACCAGCAGCAACAATCAG	NM_001402.5	translation. peptide chain elongation	1.99 (99%)
*GAPDH* (235 kb)	Glyceraldehyde 3-phosphate dehydrogenase	F: GAAGGTGAAGGTCGGAGTC R: TCGCTCCTGGAAGATGGTG	NM_002046.7	carbohydrate metabolism. glycolysis	1.97 (97%)
*HPRT1* (230 kb)	Hypoxanthine-guanine phosphoribosyltransferase	F: TGCTGACCTGCTGGATTACA R: GCCTGACCAAGGAAAGCAAAG	NM_000194.3	transferase. purine metabolism	2.08 (108%)
*PPIA* (173 kb)	Peptidylprolyl isomerase A	F: TCTGAGCACTGGAGAGAAAGG R: CAGGACCCGTATGCTTTAGG	NM_021130.4	protein folding. signal transduction. transcription	1.97 (97%)
*RPL13A* (151 kb)	Ribosomal protein L13a	F: GCAAGCGGATGAACACCAAC R: GGGATGCCGTCAAACACCTT	NM_012423.4	translation	1.95 (95%)
*RPLP0* (163 kb)	Ribosomal protein. large. P0	F: ATGGCAGCATCTACAACCCT R: AGGACTCGTTTGTACCCGTT	NM_053275.4	translation	2.03 (103%)
*SDHA* (205 kb)	Succinate dehydrogenase complex. subunit A	F: TGTTGCAAGAAGGTTGTGGG R: ACCTTGTAGTCTTCCCTGGC	NM_001294332.2	succinate dehydrogenase activity. metabolism	1.87 (87%)
*TBP* (206 kb)	TATA-binding protein	F: AGGGATTCAGGAAGACGACG R: CAAATAATGCCCCTTCCCGG	NM_003194.5	transcription factor	1.92 (92%)
*YWHAZ* (231 kb)	Tyrosine 3-mono-oxygenase/tryptophan 5-monooxygenase activation protein zeta	F: TGGCGGGGAATAAAAGGGAT R: ACCGTTTCTGCCCTTATCCA	NM_001135701.2	signal transduction	1.99 (99%)
*target genes*					
*MMP1* (120 kb)	matrix metalloproteinase 1	F: GGAAGCCATCACTTACCTTGC R: TCTAGAGTCGCTGGGAAGCTG	NM_002421.4	extra cellular matrix	2.20 (120%)
*MMP3* (104 kb)	matrix metalloproteinase 3	F: TGGGCCAGGGATTAATGGAG R: GGCCAATTTCATGAGCAGCA	NM_002422.5	extra cellular matrix	2.04 (104%)

### RNA quality, primer efficiency and technical viability

In order to evaluate the quality of the RNA used as sample material, the optical density absorption ratios A260/A280 were first measured using the NanoDrop 2000c (Thermo Fisher Scientific). The RNA analysed in this way showed an average ratio of 2.06 (± 0.06), which indicates a high purity of the isolated RNA. In addition, the RIN (RNA Integrity Number) value was determined for each individual RNA sample. These values ranged from 4.7 to 8.1, indicating intact RNA. A RIN value above 5.5 was previously defined as a meaningful threshold; however^51^, even moderately degraded samples with a degradation pattern can yield an acceptable qRT-PCR expression profile^52^.

The efficiency of gene amplification was calculated as described in the next section, with results ranging from 1.87 to 2.20, corresponding to an efficiency range of 87–120% (Table 3).

### Data analysis and determination of gene stability

To validate the quality or stability of each of the preselected genes as the most suitable for normalization, the expression profiles of 10 different reference genes were analysed based on different statistical models. As a basis for all calculations, the threshold cycles (Ct) subtracted from the baseline (Corbett Rotor-Gene 6000 software) of three intra-assay samples were averaged and analysed. The efficiency of each primer pair analysed was calculated using a linear regression of the Ct values of a standard dilution series over the slope according to the method of Pfaffl 2001^53^.

To determine the stability of the reference genes the Ct values of each sample were used to calculate stability using four various evaluation algorithms: (1) NormFinder (https://www.moma.dk/software/NormFinder) determines a stability value (S) by estimating the variance of gene expression of each possible gene within and between the study groups. The stability value (S) a value close to ‘0’ indicates high stability. Analysis of the total sample set showed that all examined candidate genes displayed an S value of loss than 1 and could therefore be considered stable^10^, (2) geNorm (https://genorm.cmgg.be/) analyses the stability (M-value) for each gene by measuring the variation with all other genes. By repeatedly excluding the least stable genes, the most stable gene pair is determined^11^, (3) the ΔCt method calculates the ΔCt values of the genes in every possible combination. By determining the average variation, the gene with the lowest variation can be identified^12^, and (4) Pearson correlation analysis compares the gene expression of one gene with the average expression of all other candidate genes. For this purpose, the expression levels were determined by log-quadratic transformation of the relative expression value R and plotted against the mean Ct value of the entire transcript set. Gene stability was determined by linear regression analysis. The coefficient of determination (r^2^) was calculated to validate the ability of each individual transcript to estimate the expression value of the others. The coefficient of determination indicates the extent to which the data representing expression corresponds to the expression of the other reference genes under certain conditions.

To evaluate the most stable gene, the ranking of the reference genes of each algorithm was performed following the method implemented by Cheng et al.^13^(Fig. 1a).

The efficiency-based ΔΔCt method was used to calculate the normalised gene expression (R_norm_) of the target genes^53^. In addition to the different amplification efficiencies, this method also took into account the reference to a control group (for the different subgroups: male, ≤ 61 years, < 30, synovitis score 2–3).

### Statistical analysis

The efficiency-based ratio between the different subgroups was calculated based on the Ct values. SPSS 22.0 for Windows was used for statistical analysis. The statistics of gene expression ratio differences were assessed using the Mann-Whitney U test. Data are expressed as arithmetic mean (± SD) unless otherwise stated. To determine the sample distribution in the subgroups, the Fisher’s exact test (exact Chi^2^ test) was used due to the expected small sample sizes. P values ≤ 0.05 were considered statistically significant.

## Data Availability

The datasets generated and/or analysed during the current study are available in the Zenodo repository, https://doi.org/10.5281/zenodo.21258585.
